# Long-term cervical cancer survivors on disability pension: a subgroup in need of attention from health care providers

**DOI:** 10.1007/s11764-020-00877-9

**Published:** 2020-04-11

**Authors:** Alv A. Dahl, Anne Gry Bentzen, Sophie D. Fosså, Siri Lothe Hess, Rita Steen, Cecilie E. Kiserud

**Affiliations:** 1 National Advisory Unit for Late Effects After Cancer Treatment, Oslo University Hospital, The Norwegian Radium Hospital, P.O. Box 4953, Nydalen, 0424 Oslo, Norway; 2 Faculty of Medicine, University of Oslo, Oslo, Norway; 3 Department of Oncology, University Hospital of Northern Norway, Tromsø, Norway; 4 Department of Oncology, Oslo University Hospital, Oslo, Norway; 5 Department of Pain Management and Research, Oslo University Hospital, Oslo, Norway

**Keywords:** Cervical cancer, Disability pension, Long-term cancer survivors, Paid work

## Abstract

**Purpose:**

Survivors of cervical cancer have an increased risk for permanently reduced work ability qualifying for disability pension (DP). Few studies describe the social and health situation of long-term survivors of cervical cancer (LSCCs) on DP as a subgroup among LSCCs. The purpose was to investigate the socio-demographic and health status of LSCCs holding DP in a population-based cohort using LSCCs holding paid work as reference.

**Methods:**

Altogether, 354 LSCCs under 67 years (age of retirement pension in Norway) at survey participated in this study. They responded to a mailed questionnaire containing social, health, and clinical issues.

**Results:**

Among LSCCs 24% held DP at a median of 11 years (range 6–15) after diagnosis versus 12% in the general female population. Compared to LSCCs in paid work, those on DP had significantly higher mean age at survey, short education, more comorbid somatic diseases, poorer self-rated health, higher level of neurotoxic side effects, more chronic fatigue, and higher mean levels of anxiety and depression. Increased age, presence of musculo-skeletal diseases, and increased levels of depression and pain remained significantly associated with DP in multivariate analysis.

**Conclusions:**

One in four LSCCs held DP which was twice the rate of the general female population. Several somatic and psychological conditions amenable to treatment were significantly associated with holding DP.

**Implications for Cancer Survivors:**

LSCCs holding DP should check their health regularly since conditions that can be treated are common, and health care providers should be aware of this opportunity.

## Introduction

Most Western societies have disability pension (DP) for members of their working population who permanently lose their working capacity due to illness or injuries. In Norway, persons are entitled to DP if their work capacity is permanently reduced with 50% or more for any kind of work. Such persons may hold a minor paid job in addition to increase their income. In spite of that, holding DP represents a considerable reduction of income since it is 66% of the mean of annual income from the best three of the last 5 years before falling ill. If the work ability improves, DP allowance can be reversed, so the DP system is dynamic and flexible [1]. By March 2019, 200,000 (12%) of Norwegian women within working age (18 to 67 years) held DP. The main medical reasons for DP were musculo-skeletal diseases (34%) and mental disorders (32%), while cancer represented only 3% (approximately 5150 women) [1]. Reporting new cases of cancer is obligatory according to Norwegian legislation, and the annual report of the Cancer Registry of Norway (CRN) shows 316 new cases of cervical cancer nationally in 2017 [2]. At the end of that year, 7297 women were survivors of cervical cancer, and 5816 (80%) of them had survived for 5 years or more (LSCCs). Among a total of 142,567 female cancer survivors, LSCCs represented only 4.1% of them [2]. Further calculations showed that 211 of the women on DP due to cancer were LSCCs, representing a quite small number. The number of LSCCs on DP for other medical reasons than cervical cancer is unknown to us. 

For the last few years, the annual increase of Norwegian adults holding DP has been 4%. The government expresses worries for the expanding DP budget and has restricted the terms for granting DP. Persons on DP are exposed to some social stigmatization for not working and being unproductive, living on the costs of the working population [3, 4]. Besides reduced income, those on DP have lost other positive effects of staying at work: network of colleagues, use and development of competence, positive identity, and social respect. Additionally, women with cervical cancer in general are exposed to stigma related to their human papilloma infection and sexual activity, although not so much as patients with lung cancer [5, 6]. These negative attitudes could reduce health-related quality of life (HRQoL) of LSCCs on DP.

Studies of long-term cancer survivors in general have so far mostly focused on diagnostic groups [7] or on various survivorship problems [8, 9]. Two Scandinavian registry studies [10, 11] reported increased risk of DP among LSCCs compared to the general female population. We hardly know of clinical studies examining vulnerable subgroups among long-term survivors in particular, such as LSCCs on DP. Therefore, this cross-sectional questionnaire study of a Norwegian clinical cohort of LSCCs had two aims: (1) to investigate the prevalence of DP among LSCCs at survey and to relate it to normative data and (2) to compare LSCCs on DP at survey with LSCCs holding paid work on social, mental, and clinical variables as well as HRQoL. Our hypotheses were that the prevalence of DP among LSCCs would be significantly higher than norm data and that LSCCs on DP would be worse of on selected social and medical variables compared to LSCCs holding paid work.

## Material and methods

### Sampling

The CRN identified all LSCCs diagnosed between January 1, 2000, and December 31, 2007, and treated at hospitals located in the Health Regions of South-Eastern Norway (2.8 million inhabitants) and of Northern Norway (0.5 million inhabitants). LSCCs were included in the main study if they were alive, aged ≤ 75 years, had no history of second cancer, were considered tumor-free, and not on any cancer treatment as of December 31, 2012. Co-operating gynecologists responsible for the management of these LSCCs at the relevant hospitals approved that the survivors were contacted by mail. The recruitment and selection of LSCCs for this sub-study on DP is shown in Table 1, and our study sample consists of 354 LSCCs.

**Table 1:** Recruitment and selection of long-term cervical cancer survivors for this study

Long-term cervical cancer survivors	Region South-East *N* (%)	Region North *N* (%)	Total sample *N* (%)
Identified and invited	819	146	965
Return due to unidentified addresses	8	3	11
Valid invited	811 (100)	143 (100)	954 (100)
Non-responders to the survey	279 (34)	51 (36)	330 (35)
Responders to the study	532 (66)	92 (64)	624 (65)
Responders with valid questionnaires	461 (57)	85 (59)	546 (57)
Excluded responders with valid questionnaires			
Age ≥ 67 years at survey (retired)	63	3	66
Relapse of cervical cancer	49	12	61
Not on disability pension or in paid work	48	17	65
Included in this study	301 (37)	53 (37)	354 (37)

### Treatment issues

The patients were treated according to Norwegian guidelines described in a previous publication [12] and summarized here: Patients with minimal disease (FIGO stage Ia) were treated with *conization*. Patients with disease of limited volume [FIGO stages Ib–IIa except stage Ib2 (tumor > 4 cm)] were treated with *major surgery* in terms of radical hysterectomy with pelvic lymph node dissection with or without bilateral salpingo-oophorectomy. Patients with locally advanced disease (FIGO stage IIb-IVa) were mainly treated with external-beam pelvic radiation to the tumor and the regional lymph nodes, combined with intra-cavitary radiation targeting the tumor, and concomitantly low-dose cisplatin-containing chemotherapy (*chemo-radiation*). A small sub-sample received neoadjuvant chemotherapy followed by major pelvic surgery. Another few patients had combinations of surgery and external-beam pelvic radiation along with chemotherapy. Due to small sample sizes, these two groups were merged into one group of *surgery combined* with either chemo-radiation and/or neoadjuvant chemotherapy. Patients who received treatment for recurrence were allocated to the respective treatment group depending on treatment modality.

### Occupational status

Occupational status at survey was self-reported, and the DP group consisted of LSCCs holding DP. Twenty-one LSCCs held DP at their CC diagnosis, while 64 LSCC were granted DP later on. When we compared these two groups, no significant between-group differences were observed concerning socio-demographic, treatment, or somatic and mental health variables (data not shown). We therefore combined these two groups further in the study. The paid work group (reference) included LSCCs working part-time or full-time as employees or being self-employed.

### Scales

#### The Fatigue Questionnaire

 The fatigue questionnaire (FQ) measures fatigue severity and contains questions concerning mental- (4 items) and physical fatigue (7 items) for the last 4 weeks. Each item is rated from 0 (as before) to 3 (very much worse). The mental fatigue score ranges from 0 to 12 and the physical score from 0 to 21, with higher scores signifying more fatigue [13]. An additional item covers the duration of the fatigue experience with the response alternative being “6 months or more”. Concerning chronic fatigue, a dichotomized score for each response alternative (0=0, 1=0, 2=1, 3=1) was used, and chronic fatigue was defined as a dichotomized sum score of ≥ 4 with duration of ≥ 6 months [13]. Internal consistencies measured by Cronbach’s coefficient alphas were 0.88 for physical and 0.68 for mental fatigue. 

#### The Hospital Anxiety and Depression Scale (HADS).

The HADS comprises 7 items each on the anxiety and depression sub-scales rated for last week. The item scores range from 0 (not present) to 3 (highly present), so the sub-scale scores range from 0 (low) to 21 (high). Only the anxiety subscale was adopted, and Cronbach’s alpha was 0.86 [14].

#### The Patient Health Questionnaire-9

The patient health Questionnaire-9 (PHQ-9) contains 9 items covering depression for the last 2 weeks, and each item is scored from 0 (not at all) to 3 (nearly every day), providing a 0 to 27 severity score. Alpha was 0.86 [15].

#### The European Organization for Research and Treatment of Cancer (EORTC) QLQ-C30 version 3

 evaluates HRQoL by rating functions and symptoms using multi-item scales: 5 functional dimensions and 9 symptom scales, and a general health and overall quality of life item. All ratings are transformed to 0–100 scales, where higher scores on the functional dimensions indicated better function and higher symptom scores indicated more symptoms [16]. Regarding the functional dimensions and global QoL, alphas were between 0.66 and 0.94.

#### The EORTC QLQ-CX24

 covers 24 items concerning HRQoL related to cervical cancer, and consists of 3 subscales: symptom experience, body image, and sexual/vaginal functioning if sexually active, and 5 single-item scales: lymphedema, peripheral neuropathy, menopausal symptoms, sexual worry, and sexual activity. All ratings are transformed to a 0–100 scale, where higher scores on the functional dimensions indicated a better QoL and higher symptom scores indicated more symptoms [17]. For the subscales, alpha was 0.67 for symptom experiences, and 0.86 for body image.

#### The scale for chemotherapy-induced long-term neurotoxicity (SCIN)

 contains 6 items concerning neuropathy, Raynaud’s phenomena and ototoxicity, and each item is scored from 0 (not at all) to 3 (very much). The item scores are summarized. Alpha was 0.71 [18]. 

#### Current work ability

 was compared to the lifetime best on a continuous 10-point scale from zero (“Currently not able to do work”) to 10 (“Work ability as previous life-time best”) from the *work ability index* (WAI) instrument [19, 20].

### Other variables

*Socio-demographic* Current paired relation was rated as present or absent. Level of education was dichotomized into short (≤ 12 years) and long (> 12 years). *Cardiovascular diseases* included self-report of myocardial infarction, angina pectoris, heart failure, stroke, diabetes, or hypertension. *Musculo-skeletal diseases* included self-report of arthrosis, osteoporosis, rheumatic, and other chronic diseases in muscles and/or joints. *Self-rated health* had 5 response alternatives and was dichotomized into good (excellent/very good/good) and poor (fair/poor). *Life-style issues:* Daily smoking concerned any number of cigarettes. BMI was calculated as weight in kilos/(height in meters)^2^, and obesity was defined as BMI ≥ 30. *Cancer-related:* histology, FIGO stages, treatment modalities (described above), and relapses were retrieved from the patients’ medical records.

### Statistical analyses

Between-group comparisons of continuous variables were performed with *t* tests, and in case of skewed distributions, Mann-Whitney *U* tests were used. Comparisons of categorical variables were performed with chi-square tests. These comparisons were all adjusted for age at survey, since the DP group had significantly higher mean age at survey than the paid work group. The internal consistencies of instruments were examined with Cronbach’s coefficient alpha. Associations between independent variables and DP as dependent variable (paid work as reference) were examined with bivariate and multivariable logistic regression analyses. The strength of associations was expressed as odds ratios (ORs) with 95% confidence intervals as appropriate (95% CI). Variables included in the multivariable analysis were tested for multicollinearity. Due to the size of the DP group (*N* = 85), only age at survey and seven other variables considered relevant for intervention were entered into the multivariable analysis. The *p* value was set as < 0.05, and all tests were two-sided. The statistical software applied was SPSS version 24 for PC (IBM Corporation, Armonk, New York, USA).

## Results

### Description of the sample

Among the 354 included LSCCs, median age at diagnosis was 39 years (range 24–58), median age at survey was 50 years (range 33–67), and median time from diagnosis to survey 11 years (range 6–15). Further, 52% had short education, and 72% lived in paired relationships. Twenty-two percent of LSCCs were treated with minimal invasive surgery (conization), 50% with major surgery, 17% with chemo-radiation, and 11% with major surgery combined with chemo-radiation and/or chemotherapy. Supplementary data on the total sample is reported in Tables 2 and 3.

**Table 2:** Characteristics of long-term cervical cancer survivors holding disability pension versus being in paid work at survey

Variables	Disability pension (*N* = 85)	Paid work (*N* = 269)	*p* value	Total sample (*N* = 354)
Age at diagnosis (years), median (range)	44 (27–58)	38 (24–57)	< 0.001	39 (24–58)
Age at survey (years), median (range)	56 (39–67)	49 (33–67)	< 0.001	50 (33–67)
Time diagnosis to survey, median (range)	11 (6–15)	11 (6–15)	0.14	11 (6–15)
Treatment modalities, *N* (%)			< 0.001*	
Conization	4 (5)	72 (27)	< 0.001	76 (22)
Major surgery only	45 (53)	131 (49)	0.53	176 (50)
Chemo-radiation	27 (32)	35 (13)	0.002	62 (17)
Major surgery combined	9 (10)	31 (11)	1.00	40 (11)
Neurotoxic side effects, mean (SD)#	5.5 (3.8)	3.1 (3.2)	0.001*	3.6 (3.4)
Short education, *N* (%)	64 (76)	118 (44)	< 0.001*	182 (52)
In paired relationship, *N* (%)	54 (64)	199 (75)	0.049*	253 (72)
Work ability at survey, mean (SD)#	3.2 (2.9)	8.8 (1.7)	< 0.001*	7.5 (3.0)
Cardiovascular disease, *N* (%)	30 (35)	37 (14)	< 0.001*	67 (19)
Musculo-skeletal diseases, N (%)	44 (52)	52 (19)	0.001*	96 (27)
Poor self-rated health, *N* (%)	48 (57)	27 (10)	< 0.001*	75 (21)
Obesity, *N* (%)	15 (19)	34 (13)	0.05*	49 (14)
Daily smoking, *N* (%)	22 (26)	44 (16)	0.22*	66 (19)
HADS-Anxiety, mean (SD)#	8.1 (3.4)	6.6 (2.8)	< 0.001*	7.0 (3.0)
PHQ-9 Depression, mean (SD)#	8.5 (5.5)	4.7 (3.7)	< 0.001*	5.6 (4.6)
Chronic fatigue, *N* (%)	31 (37)	50 (19)	< 0.001*	81 (23)

#Non-parametric Mann-Whitney test; *adjusted for age at survey

**Table 3:** Health-related quality of life measures in long-term cervical cancer survivors holding disability pension versus being in paid work at survey

Variables	Disability pension (*N* = 85)	Paid work (*N* = 269)	*p* value	Total sample (*N* = 354)
EORTC functions, mean (SD)				
Physical function	71.3 (19.4)	92.1 (12.5)	< 0.001*	87.3 (16.8)
Role function	62.5 (31.5)	89.6 (20.8)	< 0.001*	83.4 (26.0)
Emotional function	68.6 (25.6)	84.6 (18.6)	< 0.001*	80.8 (21.6)
Cognitive function	66.3 (27.0)	86.8 (17.9)	< 0.001*	82.0 (22.3)
Social function	57.3 (29.3)	86.2 (21.3)	< 0.001*	79.2 (26.5)
Global quality of life	53.7 (23.8)	77.7 (19.1)	< 0.001*	72.0 (22.7)
EORTC symptoms, mean (SD)				
Fatigue	47.6 (26.6)	24.9 (22.4)	< 0.001*	30.3 (25.4)
Nausea	8.9 (17.8)	3.8 (11.1)	0.001*	5.1 (13.4)
Pain	47.3 (33.4)	16.9 (25.3)	< 0.001*	23.6 (29.5)
Dyspnea	24.5 (31.3)	11. 3 (21.6)	< 0.001*	14.1 (24.5)
Sleep problems	49.6 (34.5)	26.9 (27.3)	< 0.001*	32.6 (30.7)
Appetite	20.5 (28.4)	5.7 (14.7)	< 0.001*	9.3 (20.0)
Constipation	22.1 (32.2)	9.9 (20.8)	< 0.001*	12.7 (24.7)
Diarrhea	30.9 (35.2)	15.0 (25.5)	< 0.001*	18.7 (28.2)
Financial problems	32.1 (31.2)	4.5 (13.4)	< 0.001*	10.9 (22.1)
Cervical cancer HRQoL (CX24)				
Symptom experience	22.4 (13.3)	12.7 (10.2)	< 0.001*	14.9 (11.6)
Body image	31.8 (27.8)	19.0 (21.8)	< 0.001*	22.0 (24.0)
Lymphedema	39.7 (45.8)	24.0 (30.0)	<0.001*	28.1 (35.2)
Peripheral neuropathy	43.1 (34.4)	20.8 (27.4)	< 0.001*	26.4 (30.6)
Menopausal symptoms	39.8 (35.9)	26.0 (34.1)	0.003*	29.1 (35.0)
Sexual worry	24.1 (34.3)	12.6 (24.7)	0.005*	15.7 (28.0)
Sexual activity*	30.2 (29.1)	38.2 (32.5)	0.34*	36.5 (32.0)

*Non-parametric Mann-Whitney *U* test

### Disability pension (DP)

The prevalence of DP among LSCCs was 24% (95%CI 20–29%) which is significantly higher than the prevalence of 12% among Norwegian women aged 18 to 67 years (*p* < 0.001). Twenty-one LSCCs (25%) held DP at their CC diagnosis, while 64 LSCCs (75%) were granted DP later on, but as indicated in the Methods section these LSCCs were merged since the between-group comparisons showed no significant differences.

### Comparisons of the DP and the paid work groups

The DP group had a significantly higher mean age both at diagnosis and at survey, and therefore, other between-group comparisons were adjusted for age at survey. Concerning type of treatment, the DP group had a significantly lower proportion of LSCCs treated with conization and a higher proportion of treated with chemo-radiation than the paid work group. In concordance with this finding, the level of neurotoxic side effects was significantly higher among LSCCs holding DP. As to social variables, LSCCs holding DP more frequently had short education, and their mean work ability score was significantly lower than among LSCCs in paid work. The proportions with poor self-rated health, and comorbid cardiovascular and musculo-skeletal diseases were significantly higher in LSCCs on DP than among those in paid work. The mean scores of anxiety and depression and the proportion with chronic fatigue were all significantly higher among LSCCs holding DP (Table 2). All EORTC QLQ C-30 functions and global quality of life scores were significantly lower in LSCCs holding DP, while all the symptom scores were significantly higher (Table 3). Concerning the EORTC CX24, the symptom experience scores were significantly higher in women with DP, while the mean sexual activity score was significantly lower compared to women holding paid work (Table 3).

### Bivariate and multivariable regression analyses

In bivariate analyses older age at survey, comorbid cardiovascular and musculo-skeletal diseases, having chronic fatigue, sleep problems, increased levels of depression, pain, and lymphedema were significantly associated with holding DP at survey (Table 4). In the multivariate analysis age at survey, comorbid musculo-skeletal diseases, and increased levels of pain and depression remained significantly associated with holding DP (Table 4).

**Table 4:** Logistic regression analyses of independent variables and being on disability pension (*N* = 85) and in paid work (*N* = 269) (reference) at survey

Variables	Univariate analyses			Multivariable analysis		
	OR	95%CI	*p*	OR	95%CI	*p*
Age at survey	1.09	1.05–1.13	< 0.001	1.11	1.06–1.16	< 0.001
Cardiovascular disease	3.42	1.95–6.01	< 0.001	1.62	0.81–3.26	0.17
Musculo-skeletal disease	4.48	2.66–7.55	< 0.001	2.74	1.47–5.11	0.002
Chronic fatigue	2.58	1.50–4.42	< 0.001	1.09	0.48–2.47	0.85
PHQ-9 Depression	1.20	1.13–1.28	< 0.001	1.17	1.07–1.29	0.001
EORTC QLQ-30 Pain	1.03	1.02–1.04	< 0.001	1.02	1.01–1.03	0.008
EORTC QLQ-30 Sleep	1.02	1.01–1.03	< 0.001	1.00	0.99–1.01	0.56
EORTC CX24 Lymphedema	1.01	1.01–1.02	0.001	1.00	0.99–1.01	0.78

## Discussion

In our sample at median of 11 years after diagnosis, aged median 50 years, 24% of our LSCCs reported holding DP at survey. This prevalence was twice as high as the rate DP among Norwegian females. Compared to LSCCs in paid work, the DP group had significantly older mean age at survey and had shorter education. The DP group had less often conization and more chemo-radiation and accordingly a higher level of neurotoxic side effects. The DP group had more often comorbidity, poor health, and chronic fatigue. The levels of anxiety, depression, and EORTC symptom scales were significantly worse in the DP group. The EORTC function scales and cervix-related issues (CX24) mean scores were all significantly lower in the DP group. In multivariate analysis age at survey, comorbid musculo-skeletal diseases, and increased levels of pain and depression remained significantly associated with holding DP. Like in the two Scandinavian registry studies [10, 11] we observed increased risk of DP among LSCCs compared to the general female population. More intensive treatment regimens were significantly associated with increased prevalence of DP (Table 2), confirming previous Swedish findings among LSCCs [11]. In contrast to that study, we found the demographic factors of older age and shorter education to be significantly associated with DP. As in studies of the general female population, our study of LSCCs replicated short education, poor self-rated health, comorbidity, and increased levels of mental distress as factors associated with holding DP [4, 21]. We also confirmed the relevance of somatic comorbidities reported in previous registry studies of DP among female breast cancer survivors [22, 23]. As in other clinical studies [24, 25] we observed significant associations with anxiety/depression, fatigue, and more intensive treatment. Generally, many studies of long-term cancer survivors have demonstrated high levels of adverse effects, or have identified subgroups with different levels of adverse effects [8, 9]. We observed higher level of neurotoxic adverse effects and higher rate if chronic fatigue in the DP group. The LSCCs on DP showed increased prevalence of comorbidity; higher levels of current depression, anxiety, and pain; and more lymphedema than the paid work group. Some of these findings should eventually be amenable to therapy eventually improving the health status and HRQoL of the LSCCs. This is especially important since holding DP among LSCCs was positively associated with increased mortality rate [4].

We have demonstrated that LSCCs on DP represent a subgroup with multiple of somatic and mental health problems, and most probably poorer economy compared to LSCCs in paid work. These differences are reflected in poorer scores of the DP group on all general HRQoL functions and symptoms (EORTC QLQ C30) as well as poorer scores on the cancer-specific HRQoL (EORTC QLQ-CX24). The cancer-specific HRQoL concerns many specific gynecological and sexual problems. We tested lymphedema in our multivariate analysis, but the association was not significant competing with comorbidity, pain, and depression.

Mehnert et al. [26] introduced a complex model for the relationship between cancer survivorship and work outcomes such as DP. Among individual and interpersonal factors, we confirmed that short education is an important determinant of DP independent of the cervical cancer trajectory. Poor self-reported health, somatic comorbidity, and reduced HRQoL in the group of LSCCs are all factors that could have been established long before our survey. All of them could contribute to reduced working capacity in addition to cervical cancer, and eventually, they could have contributed to a status qualifying for DP. An alternative explanation could be that getting DP frequently means permanent omission from work life leading to reduced HRQoL. Our cross-sectional study cannot sort out the time line concerning these matters. In Norway, holding DP is a reversible process in case of improved health and working capacity. Treatment of associated factors like anxiety/depression, comorbidity, pain, and insomnia may improve and probably increase working capacity and/or HRQoL among LSCCs holding DP. These are all ailments that should have been addressed during the sick leave (up to 1 year) and the work assessment allowance period (up to 3 years) generally preceding DP. But they can also be addressed and eventually treated after granting of DP. The LSCCs should therefore be conscious about their health, and the health care providers responsible for LSCCs should keep an active attitude toward treatable conditions. In cancer survivorship research, it is often relevant to consider the findings of other approaches to the issue under study. In this regard, aspects of DP have been intensively studied by social security physicians and sociologists, and the terms of DP are to somewhat commensurable in all the Nordic countries. A review of such studies [4] showed the following variables were associated with holding DP: older age, being female, non-paired civil status, short education, poor somatic health, increased depression or anxiety, obesity, and daily smoking. In our study, most of these risk factors were supported except for civil status and life style variables like obesity and smoking.

An advantage of our study is the considerable sample size giving enough statistical power to the group of women holding DP, which implies more detailed description of clinical variables relevant for the understanding of the health status of LSCCs holding DP. Another strength is our use of well-established self-rating instruments with adequate psychometric properties. Our overall response rate of 57% at a mean of 11 years post diagnosis must also be considered as acceptable. However, our lack of data for doing an attrition analysis trying to characterize the non-respondents further must be considered a weakness. Another limitation is our lack of data concerning the medical reasons for DP. Our questionnaire also lacked a scale concerning experienced stigma related to cervical cancer and DP. Since we only have cross-sectional data, we present associations between variables, rather than causal findings.

## Conclusion

Close to one in four LSCCs were holding DP at survey, in contrast to 12% of women in the general population. LSCCs on DP were worse off on somatic and mental health as well as on general and cancer-specific HRQoL compared to LSCCs in paid work. Some of the factors associated with DP are amenable to identification and treatment, which calls for an active attitude of both LSCCs and health care providers in order to improve their health, HRQoL, and eventually their work ability. Our hypothesis of LSCCs holding DP as a subgroup with multiple problems was supported.
